# Optical mapping as a routine tool for bacterial genome sequence finishing

**DOI:** 10.1186/1471-2164-8-321

**Published:** 2007-09-14

**Authors:** Phil Latreille, Stacie Norton, Barry S Goldman, John Henkhaus, Nancy Miller, Brad Barbazuk, Helge B Bode, Creg Darby, Zijin Du, Steve Forst, Sophie Gaudriault, Brad Goodner, Heidi Goodrich-Blair, Steven Slater

**Affiliations:** 1 Monsanto Company, 800 North Lindbergh Boulevard St. Louis, MO 63167, USA; 2 OpGen Technologies, Inc., 510 Charmany Drive, Suite 151, Madison, WI 53719, USA; 3 Donald Danforth Plant Sciences Center, 975 North Warson Road St. Louis, MO 63132, USA; 4 Institut für Pharmazeutische Biotechnologie, Universität des Saarlandes, 66123 Saarbrücken, Germany; 5 University of California, San Francisco, Department of Cell and Tissue Biology, San Francisco, CA 94143, USA; 6 University of Wisconsin, Milwaukee, Department of Biological Sciences, Milwaukee, WI 53211, USA; 7 Institut National de la Recherche Agronomique-Université de Montpellier II, 34095 Montpellier, France; 8 Hiram College, Department of Biology, Hiram, OH 44234, USA; 9 University of Wisconsin, Department of Bacteriology, Madison, WI 53076, USA; 10 Arizona State University, The Biodesign Institute and Department of Applied Biological Sciences, 7001 E. Williams Field Road, Mesa, AZ 85212, USA

## Abstract

**Background:**

In sequencing the genomes of two *Xenorhabdus *species, we encountered a large number of sequence repeats and assembly anomalies that stalled finishing efforts. This included a stretch of about 12 Kb that is over 99.9% identical between the plasmid and chromosome of *X. nematophila*.

**Results:**

Whole genome restriction maps of the sequenced strains were produced through optical mapping technology. These maps allowed rapid resolution of sequence assembly problems, permitted closing of the genome, and allowed correction of a large inversion in a genome assembly that we had considered finished.

**Conclusion:**

Our experience suggests that routine use of optical mapping in bacterial genome sequence finishing is warranted. When combined with data produced through 454 sequencing, an optical map can rapidly and inexpensively generate an ordered and oriented set of contigs to produce a nearly complete genome sequence assembly.

## Background

*Xenorhabdus *species are symbiotic bacteria associated with insectivorous nematodes of the genus *Steinernema *(for review see [1]) They reside in a specialized segment of the nematode gut [2,3], and provide insecticidal proteins [4,5] and small molecules [6-10] that help to kill the insect larvae that are the prey of the nematode. Both organisms reproduce in the dead larvae, the *Xenorhabdus *colonize the young nematodes, and the cycle repeats [11]. *Xenorhabdus *are closely related to the enteric gamma proteobacteria such as *Escherichia coli *[12], and are an emerging model for both mutualism and pathogenicity in invertebrate hosts. To better understand the genetic basis of these relationships, we are sequencing the genomes of two *Xenorhabdus *species: *X. nematophila *ATCC 19061 and an *X. bovienii *strain from Monsanto's collection.

In the course of this work, we found that the *X. nematophila *genome contained large numbers of highly repetitive DNA regions, and efforts to finish the genome stalled. We sought a means to produce whole-genome maps for comparison with the genomic DNA sequence, and identified optical mapping as a useful means to align and orient the genome sections *in silico*. In addition, we produced an optical map of a second genome that we had considered finished, and identified a large sequence inversion that would have otherwise been unnoticed.

## Results

### A whole-genome restriction map permits finishing of the *X. nematophila *genome sequence

Eight-fold genome sequence coverage of *X. nematophila *ATCC19061 (Goodrich-Blair et al, in preparation) was generated, with 26,976 reads from a 2–4 kb insert library and 41,376 reads from a 4–8 kb insert library. This yielded an initial assembly consisting of 100 contiguous sequences (contigs) greater than 2 kb, 14 contigs greater than 100 kb, and 2 contigs greater than 200 kb length. Our initial research had shown the presence of a 150 Kb plasmid in addition to the circular chromosome (Goodrich-Blair and Goodner, unpublished).

It became rapidly clear that multiple areas of repeated sequence were causing problems. In fact, the final *X. nematophila *sequence assembly shows a nearly identical 12 Kb region found on both the plasmid and chromosome, many transposons (including over 30 copies of a single transposon) scattered throughout the genome, and seven rRNA regions. Using the paired clone-end sequences and syntenic comparison to the related species *Photorhabdus luminescens *[13], resolution of misassembles and gap closure was attempted by walking across individual clones and amplifying potentially adjacent regions using the polymerase chain reaction (PCR). The resulting assembly contained over 50 contigs, but most lacked linkage information from gap-spanning paired ends. Multiplex PCR resolved some gaps, but provided no indication about whether the amplified product was actually the correct size, whether a particular gap was resistant to amplification, or whether a reaction failed because the primers were not properly paired to cross a gap. After four months of concerted effort, the assembly still contained 36 contigs which collectively contained several hundred copies of transposons plus seven ribosomal RNA coding regions. Given this complexity, optical mapping was attempted to provide a structural scaffold for aligning and orienting the contigs.

Optical mapping permits assembly of whole-genome restriction endonuclease maps by digesting immobilized DNA molecules and determining the size and order of fragments [14-22]. In collaboration with OpGen Technologies (Madison, WI), optical maps of *X. nematophila *ATCC19061 were produced using *AflII *and *EagI *restriction enzymes. Through repeated overlapping of restriction maps from individual molecules (over 50-fold coverage), OpGen's assembler program reconstructed the ordered restriction map of the genome [23].

**Figure 1:**
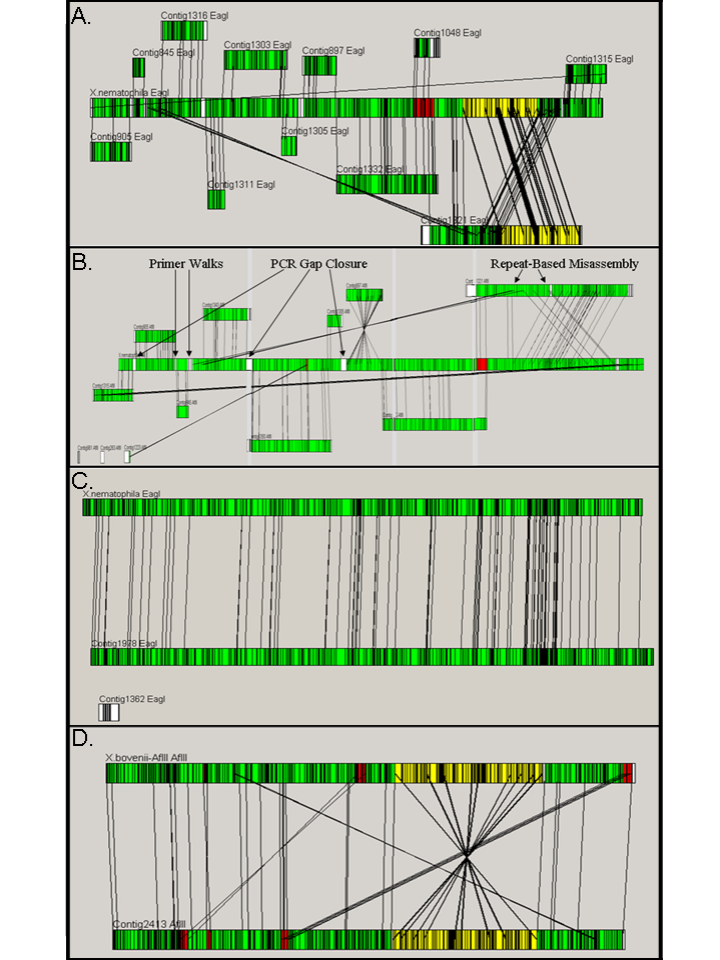
**Alignments between the whole-genome optical maps and the *in silico *genome sequence assemblies at various stages of the project**. Green regions indicate perfect alignment, white regions indicate no alignment, red regions indicate sequence that is present on at least two contigs, and yellow regions indicate inversions. Lines between maps indicate the position of identical sequences on the two maps, and can be used to visually identify misassemblies and inversions. **Panel A**: An early comparison of an optical map derived from *EagI *digestion of the *X. nematophila *genome to the assembled contigs generated by traditional sequencing technologies. All contigs could be ordered for gap closure. In addition, the optical map indicated an overlooked misassembly. **Panel B**: The finishing strategy, including gap closure and misassembly resolution, was simplified using the optical map as an assembly model. The *X. nematophila *optical map derived from an *AflIII *digestion of the chromosome is presented as a single contig in the center. The sequenced genome contains nine contigs that have a corresponding match to the optical map. The *X. nematophila *plasmid is 158 Kb and is too small to be identified using the current optical map technology. Nonetheless, small sections of the plasmid can be identified as regions that do not have corresponding optical map locations (white in figure). **Panel C**: Comparison of the final assembly of the *X. nematophila *genome (bottom) to the optical map (top) for the *EagI *digest. The non-aligned contig represents the plasmid, which was generated by traditional sequencing technologies. **Panel D**: Comparison of the finished sequence of *Xenorhabdus bovienii *to the *EagI *optical map revealed a large inverted region of the genome. The red regions indicate regions of repeats within the genome that cannot be resolved by optical mapping. These regions were resolved using traditional sequencing methods. The sequenced genome was easily re-oriented to correct the assembly.

Each restriction map produced by optical mapping was aligned with the restriction map predicted from the *X. nematophila *genome sequence. The map permitted alignment and orientation of all 36 contigs, and identification of misassemblies, allowing production of PCR products to cover all remaining gaps in the sequence (Figure 1 panel A). Once the optical map was available, PCR, sequencing, and validation of the final assembly were accomplished in approximately one month. The map also detected several regions of misassembled sequence, including a plasmid that was integrated into the chromosomal sequence among the assembled contigs (Figure 1 panel B). The plasmid shares a highly conserved stretch of sequence with the chromosome (only 37 bp differences over approximately 12.5 kb), and this duplication led to the *in silico *misassembly. The final sequenced genome aligned directly to the restriction map generated by optical mapping (Figure 1 panel C).

### Optical mapping identifies an assembly error in the *X. bovienii *sequence

In addition to *X. nematophila*, we had previously sequenced and assembled the genome of the related organism *X. bovienii *using traditional finishing technologies. Although the *X. bovienii *genome does not contain as many repeats as that of *X. nematophila*, the *X. nematophila *project had shown the value of non-sequence-based methodologies in validating sequence assemblies. After generating an optical map for *X. bovienii *(NCBI designation *Xenorhabdus bovienii *SS-2004) using *Afl*III, a large inversion was detected in the sequence assembly, permitting a simple re-orientation of the data and correction of the genome sequence (Figure 1 panel D). It is doubtful that this assembly inversion would have been detected without the optical map.

## Discussion

The *Xenorhabdus *genomes analyzed in this project contain many highly repetitive regions, and these became a major obstacle in our attempts to assemble the genome sequences. Genome finishing traditionally relies on cosmid libraries or overlapping restriction maps of BACs to build larger meta-contigs. With the *X. nematophila *genome the traditional approach failed, and we used a genome-scale restriction map generated by optical mapping. This permitted rapid and accurate closing of *X. nematophila*, and provided savings of labor, reagents and time. Finishing the *X. nematophila *genome sequence would have otherwise required production of a fine-scale genetic-physical map at much greater cost in time and materials. Optical mapping also identified an inversion in the X. bovienii genome sequence assembly that we  had considered finished.

High throughput processes like DNA sequencing normally require trade-offs among cost, speed, and data quality. Sequencing costs are being reduced, and speed increased, by novel methods such as the pyrosequencing technology of 454 Life Sciences [24,25]. However, 454 technology produces shorter sequences (100 to 250 bases per reaction) than traditional Sanger sequencing using ABI instrumentation (800–1000 bases per reaction). These shorter 454-derived sequences mean that sequence contigs are also, on average, shorter than those produced using ABI instruments. However, the lower quality of sequence assemblies from 454 data is compensated by speed and cost considerations. Excluding the cost of purchasing the instrumentation and labor, a typical 5 Mb bacterial genome takes approximately 2 days and costs about $6,000 in consumables using 454. The same genome sequence produced by ABI instrumentation would cost approximately 10-fold more and take several weeks. In our experience, a typical 5 Mb assembly using 454 data would contain about 80–90 contigs, with an average length around 60–70 Kb. A similar genome assembled using data from ABI 3730 instruments would contain about 50 contigs with an average length >100 kb. Both strategies would typically add about 4,000 end-paired sequences from cosmids or phosmids to help scaffold the genome, at a cost of about another $4,000.

The current cost for an optical map with a single enzyme is approximately $7,000, and adding a second enzyme costs around another $3,000 (in our experience, only one enzyme is typically required). The optical mapping system can accurately quantify fragments down to about 4 kb in size, and a contig of 40 kb has an approximately 80% probability of being placed within a whole genome optical map (OpGen, unpublished data). When all of these data are combined, a 454 shotgun sequence plus cosmid end sequences and an optical map, can produce an assembled and oriented set of contigs containing about 95% of the genome for under $20,000 with very limited input by a human finisher. This is about one-fifth the cost of a project produced through traditional means, provides very high quality data, and puts production of finished bacterial genomes within the reach of even small labs. We are currently working on a genome produced in this manner that will be primarily closed using undergraduate researchers supported by some bioinformatics infrastructure.

## Conclusion

Even on these relatively small genomes, the whole-genome maps were very valuable. In the *X. nematophila *project, we had the advantages of long sequence reads and clone end-pairing data, yet still were unable to assemble contigs because of the presence of numerous highly repetitive sequences. The optical map allowed rapid closure of one genome and identified an assembly error in a fully-assembled genome sequence that gave no prior indication of having errors.

As shotgun sequencing costs come down, the optical map becomes a significant portion of the budget for a new bacterial genome sequence. However, for genomes that contain particularly large numbers of repetitive sequences, require finishing, or simply require ordered and oriented contigs from shotgun sequence, an optical map can increase the speed and decrease the overall cost of the project. We also expect that mapping costs will come down as optical mapping becomes more routinely used by sequencing centers, and as resolution of fragment size moves toward the 1–2 kb range. We now routinely confirm the *in silico *assemblies of bacterial genomes using a whole-genome restriction map, and believe this is a relatively low cost method to speed finishing and ensure accuracy of finished bacterial genome sequences.

## Methods

### Genomic library construction, DNA sequencing, and finishing

The genomic DNA was sonicated at scale of 8.5 for two seconds, repeated 3 times (Missonex Inc. Sonicator XL2020). The ends were repaired using T4 DNA polymerase and T4 kinase (NEB) and fractionated on a 1% agarose gel. Fractions representing size ranges 2–4 KB and 4–8 KB were excised from the gel and purified using a Qiagen Gel Quick extraction column (Qiagen, Cat No 28704). DNA samples from the isolated fractions were checked for size on an agarose gel and then ligated into pUC18.

Clones were plated and colonies picked on a Q-Bot (Genetix), to achieve 80% of sequence from the 2–4 KB library and 20% of sequence from the 4–8 KB library. Each template was sequenced using the Big Dye terminator protocol (Applied Biosystems) and analyzed on ABI 3700 and ABI 3730 sequencers. Both the forward (M13 -40) and reverse (M13 -21) primer were used on each template, yielding two related sequences per subclone. Data were assembled using phred/phrap (ver. 0.990319; [26,27]), and finished in Consed and Autofinish (v.13.0; [28-30]) using a variety of directed primer walks on subclones, and using PCR/walking to close any gaps. The sequence assemblies were confirmed by OpGen using optical mapping, as described below and previously [14-22]. These alignments were viewed using OpGen's MapViewer software (Figure 1; see below).

### Optical map construction

Optical maps were prepared at OpGen Technologies, Inc. (Madison, WI) according to methods described previously [22,23]. Briefly, high molecular weight DNA was prepared by first embedding bacterial cells harvested at stationary phase in low melting temperature agarose plugs, followed by treatment with bacterial lysing solutions. The genomic DNA was recovered after thoroughly rinsing the plugs in TE followed by melting the plugs at 42 C and subsequent treatment with β-agarase. The high molecular weight DNA was then immobilized as individual molecules onto Optical Chips, digested with EagI or AflII restriction enzymes (New England Biolabs), fluorescently stained with YOYO-1 (Invitrogen) and positioned onto an automated fluorescent microscope system for image capture and fragment size measurement, resulting in high resolution single-molecule restriction maps. Collections of single molecule maps were then assembled to produce whole genome, ordered restriction maps.

### Sequence-to-map comparison

Comparisons between Optical maps and sequence contigs were performed as described previously [22]. Sequence FASTA files were converted to *in silico *restriction maps via the MapViewer software (OpGen Technologies, Inc.) for direct comparison to the Optical maps. Comparisons were accomplished by aligning the sequence with the Optical maps according to their restriction fragment pattern. Alignments were generated with a dynamic programming algorithm which finds the optimal location, or placement, of a sequence contig by first performing a global alignment of the sequence contig against the Optical map. Local alignment analysis were also performed where segments of the sequence contigs were compared to the Optical map.

## Competing interests

JH  is employed by OpGen Technologies, Inc., the commercial provider of  optical mapping technology.

## Authors' contributions

PL performed quality control analysis on the sequence and prepared an early draft of the manuscript. JH produced the optical maps. SN, ZD and NM performed the genome finishing work. SS assisted with data analysis and was the primary writer of the manuscript. BB ran automated annotation on the genomes and provided an HTML interface for analysis. HGB, SF and BG performed genetic and molecular analysis of the strains to confirm their identity prior to sequencing and optical mapping. HGB, SF, BG, HB, CD and SG assisted with data analysis. BSG conceived and coordinated the project and helped to write the manuscript.
